# Indocyanine green fluorescence angiography in the management of intestinal injuries following penetrating abdominal trauma: a case-control study comparing postoperative outcomes

**DOI:** 10.1097/JS9.0000000000002096

**Published:** 2024-10-22

**Authors:** Mohamed Q. Patel, Jens TF Osterkamp, Johan JP Buitendag, Timothy R. Forgan, Elmin Steyn

**Affiliations:** a Division of General Surgery, Tygerberg Academic Hospital, Cape Town, Western Cape; b Division of Surgery, Faculty of Medicine & Health Sciences, Stellenbosch University, Stellenbosch, South Africa; c Department of Surgical Gastroenterology, Rigshospitalet, University Hospital of Copenhagen, Copenhagen, Denmark

**Keywords:** anastomotic-related complications, indocyanine green, indocyanine green fluorescence angiography, penetrating abdominal trauma

## Abstract

**Background:**

The surgical management of penetrating hollow visceral injuries includes primary repair or exteriorization. Tissue perfusion at the site of gastrointestinal suture repair may be challenging to assess and is vulnerable to local energy transfer-related injury, micro- or macro-circulatory insufficiency, or splanchnic vasoconstriction for various reasons. Breakdown of suture lines can lead to potentially life-threatening complications. The intraoperative use of indocyanine green fluorescence angiography (ICG-FA) may reduce the risk of postoperative morbidity and mortality by ensuring optimal tissue perfusion at the chosen site of suture repair.

**Materials and methods:**

The authors conducted a retrospective review of the postoperative complications, length of ICU stay, and length of hospital stay in patients undergoing laparotomy, with and without ICG-FA for penetrating abdominal trauma at a Level One Trauma Center in Cape Town, South Africa.

**Results:**

One hundred patients were included in the study, of which 20 underwent laparotomy with ICG-FA, and 80 did not. The overall complication rate was significantly lower in the ICG-FA group (OR 0.336, *p*=0.0412). The anastomotic leak rates in the ICG-FA and control groups were 0% and 6.25%, respectively (*p*=0.5799). Revision surgery was required in 2 and 14 patients in the ICG-FA and control groups, respectively (OR 0.524, *p*=0.516). The mean length of stay in the hospital showed no statistical difference, 8.6 and 5.3 days for the ICG-FA and control groups, respectively (*p*=0.092). The mean length of ICU stay was 6.3 and 2.3 days for the ICG-FA and control groups, respectively (*p*=0.1642).

**Conclusion:**

Lower levels of overall postoperative complications and lower rates of revision surgery in patients undergoing laparotomy with ICG-FA are promising. Non-significant findings regarding the relationship between the usage of ICG-FA and anastomotic leak rates suggest the need for larger randomized studies.

## Introduction

Highlights

- Microperfusion of suture lines in penetrating gastrointestinal injuries is vital to avoid postoperative complications.
- Anastomotic-related complications can result in debilitating consequences, often requiring revision surgery.
- Indocyanine Green Fluorescence Angiography (ICG-FA) has been used to assess the microperfusion of anastomotic lines in elective gastrointestinal surgery.
- ICG-FA has been shown to be a feasible adjunct for the operative management of penetrating gastrointestinal injuries.
- ICG-FA use in penetrating gastrointestinal injuries shows promise to minimise postoperative complications.

Gastrointestinal injuries are common after penetrating abdominal trauma^1,2^. Stab wounds to the abdomen often follow a predictable injury trajectory and display a lower degree of extension of injury than gunshot injuries^2–4^. Despite advancements in diagnostics and treatment of abdominal gunshot wounds, high morbidity and mortality rates persist^5^.

Gastric injuries are managed with debridement and primary repair. Small bowel and colonic injuries can be managed with primary anastomosis^3,6^ or diversion via exteriorization of the proximal segment of the intestinal injury^2,6^. The management of intestinal injuries should be individualized based on the patient’s physiological state and the severity of the injury. Destructive colonic injuries are defined as defects greater than 50% of the bowel wall with concomitant devascularization of the mesentery of the colon. These injuries require segmental resection of the de-vascularized segments. Non-destructive colon injuries are defined as defects of less than 50% of the bowel wall^7^. Colon injuries should be managed based on their merits, irrespective of whether the right or left colon was injured^8^. Deciding on the appropriate surgical intervention for destructive colon injuries is often more complicated than for non-destructive colonic injuries^9–11^.

The anastomotic leak rate is variable and is reported to range between 4-27%^12,13^. Anastomotic leaks have severe consequences that will prolong the duration of hospitalization and increase morbidity rates. The mortality rate associated with anastomotic-related complications has been estimated to be 10–15%^14,15^.

Poor vascularity at the anastomotic site is a predictor of anastomotic failure^16–18^. The evaluation of intestinal perfusion with gunshot injuries can be challenging even for experienced surgeons, as shockwave injury might not be apparent on visual assessment^2,3^.

Indocyanine green (ICG) dye is a commonly used fluorescent intravenous contrast agent^19,20^. Near-infrared light is shone on the tissue under investigation, and a camera with an optical filter detects the ICG dye by fluorescence^19,21^. This allows for real-time assessment of visceral microperfusion, providing a surgeon with the opportunity to optimize their surgical plan and ensure optimal anastomotic perfusion and subsequently lower rates of anastomotic leak^20^.

The use of indocyanine green fluorescence angiography (ICG-FA) for elective surgical gastrointestinal procedures has been investigated. A prospective observational study used ICG-FA in patients with left-sided colorectal cancer, with ICG-FA altering operative decisions in 34.5% of cases (*n*=111)^22^.

It is essential to consider modalities that enhance emergency surgical techniques to reduce the risk of postoperative complications. The usage of ICG-FA has been investigated in the setting of acute mesenteric ischemia, with findings demonstrating its use as a feasible and reliable modality for observing intestinal perfusion in patients undergoing emergency surgery for acute mesenteric ischemia^23^.

We sought to describe and compare the postoperative outcomes of patients who underwent laparotomy with and without ICG-FA in the management of penetrating gastrointestinal injuries.

## Materials and methods

We conducted a retrospective review of patients who underwent laparotomy, with and without ICG-FA, for penetrating abdominal trauma at a Level One trauma center in Cape Town, South Africa. The study period was from March 2020 to March 2021.

Adult patients (18 years and older) were included in the study if they presented with gastrointestinal injuries as a result of penetrating abdominal trauma. This was compared to the outcomes of a cohort of patients who underwent laparotomy with ICG-FA enhancement, as reported by Osterkamp *et al.* ^24^. The current study is a further investigation into the concept of ICG-FA in trauma. Results were reported in line with the STROCCS criteria^25^. Patients who were pregnant, unable to provide informed consent, or had a known hypersensitivity to intravenous contrast were excluded from the study.

The on-call surgeon performed the procedure using ICG-FA. At laparotomy, the surgeon evaluated for gastrointestinal injuries and planned their surgical interventions using a marker to identify resection or debridement points. After surgical planning, ICG (5 milligrams) was administered intravenously, with a reassessment of gastrointestinal injuries. Fluorescence angiography was viewed using the SPY-PHI camera. Surgeons were allowed to revise their surgical plans. A further ICG-FA screen was done following the anastomoses to assess its perfusion. Indocyanine green dye (Verdye—Diagnostic Green GmbH, Aschheim-Dornach, Germany) costs R1725 (South African Rands—ZAR) per vial containing 25 mg.

**Figure 1:**
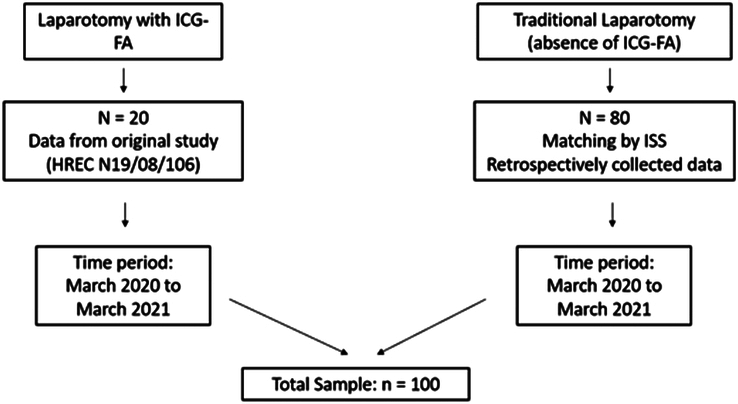
Sample size calculation. ICG-FA, indocyanine green fluorescence angiography; ISS, Injury Severity Score.

The sample size calculation was performed using Epi-Info version 7 software. We assumed a 5% level of significance (equivalent to 95% confidence intervals), a power of 80%, and a ratio of four controls to one case. Using available data on the 20 cases where 8 (40%) had complications, this value of 40% was used as the percentage of cases exposed, assuming that the complications are taken as the exposure. We expected that the intervention would reduce the rate of complications by 50%, thus the rate of complications in the controls would be doubled or 80%. This translated to an odds ratio of 0.125. The minimum sample size required was 19 cases and 75 controls, rounded off to 20 cases in the ICG-FA group and 80 controls, giving a total sample size of 100 patients (see Fig. 1).

Data for the control group were collected from various databases, namely Laboratory Services, Picture Archiving and Communication System (PACS), General Surgery Operation Registry, and Electronic patient record system.

### Statistical analysis

Physiological and anatomical injury severity scoring systems were used to describe the clinical presentation of the ICG-FA and control groups. The Injury Severity Score (ISS) is an anatomic grading system that calculates the overall severity of an injury. We used the ISS to match the ICG-FA and control groups to minimize selection bias. Quantitative variables were analyzed using medians and interquartile ranges. Mann–Whitney U-tests were used to compare the means of the two groups with data that were not normally distributed. Qualitative parameters were compared using Fisher’s exact test. Complications were compared between the two groups using Fisher’s exact test. Postoperative complications were also reported and assessed using the Clavien–Dindo classification. Statistical calculations were performed using IBM SPSS software. Statistical significance was assumed for *p* values less than 0.05.

## Results

### Demographic data

The distribution of patients by sex demonstrated a clear male predominance in both groups, with 90% of the sample in the ICG group and 97.5% in the control group (Table 1). The median age (Table 1) was 29 years (IQR 12.25) in the ICG group and 30 years (IQR 10.81) in the control group.

**Table 1:** Demographic data.

	ICG-FA	Control
Sex M, *n* (%) / F, *n* (%)	18 (90) / 2 (10)	78 (97.5) / 2 (2.5)
Age (median/IQR)	29 (12.25)	30 (10.81)
Tobacco smoking, *n* (%)	7 (35)	52 (65)
HIV positive, *n* (%)	1 (5)	1 (1)

ICG-FA, indocyanine green fluorescence angiography; IQR, interquartile range.

### Baseline characteristics at admission

Most patients had minimal to no pre-morbid conditions. Baseline clinical characteristics were described using the American Society of Anesthesiology (ASA) score, Injury Severity Score (ISS), Modified Shock Index, and Revised Trauma Score (RTS) (Table 2). The Modified Shock index was used to define the hemodynamic stability of each patient. The majority of patients presented with mild shock (Shock Index 0.6–1), 12 patients (60%) in the ICG-FA group and 42 patients (52.2%) in the control group. Only one patient (5%) in the ICG-FA group and two patients (2.5%) in the control group presented with severe shock (Shock Index >1.4). The mean ISS was 17.55 in the ICG-FA group and 15.44 in the control group (*p*=0.0529), with a maximum score of 37 in the ICG-FA group and 34 in the control group. The median RTS was 7.8408 in both groups.

**Table 2:** Severity scoring systems and risk factors.

	ICG-FA	Control	*p*
Time to surgery (h) (median/IQR)	18,30 (15.99)	14,46 (16.17)	
ASA Score (median/IQR)	1 (0)	1 (0)	
ISS (median/IQR)	13 (9)	16 (9)	0.0529
RTS (median/IQR)	7.8408 (0)	7.8408 (0)	
Modified Shock Index (median/IQR)	0.7 (0.3)	0.79 (1.0)	
No shock <0.6, *n* (%)	6 (30)	20 (25)	
Mild shock 0.6–1, *n* (%)	12 (60)	42 (52.5)	
Moderate shock 1–1.4, *n* (%)	1 (5)	16 (20)	
Severe shock >1.4, *n* (%)	1 (5)	2 (2.5)	

ASA, American Society for Anaesthesiology; ICG-FA, indocyanine green fluorescence angiography; IQR, interquartile range; ISS, Injury Severity Score; RTS, Revised Trauma Score.

### Surgical findings and interventions

Gunshot wounds are the most common mechanism of injury. In the ICG-FA and control groups, 12 patients (60%) and 56 patients (70%) sustained gunshot wounds to the abdomen, respectively. These wounds inflicted in a civilian population are likely to be low energy transfer weapons. The most common anatomic location (Table 3) of gastrointestinal tract injury was the colon, with 11 patients (26.2% of all injuries) in the ICG-FA group and 45 patients (26.5% of all injuries) in the control group. The small bowel was also a common anatomical location of injury in nine patients (21.4% of all injuries) in the ICG-FA group and 46 patients (27.1% of all injuries) in the control group. Anastomoses were undertaken in 14 (70%) and 46 (58.8%) of patients in the ICG-FA and control groups, respectively (Table 3).

**Table 3:** Surgical findings and interventions.

	ICG-FA number of injuries, *n* (%)	Control number of injuries, *n* (%)
Stomach	5 (11.9)	24 (14.1)
Duodenum	0	5 (2.9)
Small bowel	9 (21.4)	46 (27.1)
Large bowel	11 (26.2)	45 (26.5)
Mesenteric	6 (14.3)	9 (5.3)
Liver	3 (7.1%)	14 (8.2)
Gall bladder	0	2 (1.2)
Spleen	1 (2.4)	5 (2.9)
Pancreas	1 (2.4)	4 (2.4)
Kidney	2 (4.8)	5 (2.9)
Ureter	0	2 (1.2)
Diaphragm	4 (9.5)	9 (5.3)
	ICG-FA, *n* (%)	Control, *n* (%)
Anastomosis	14 (70)	47 (58.8)
Diversion	4 (20)	9 (11.3)
Anastomosis and diversion	1 (5)	12 (15)
Damage control surgery	1 (5)	12 (15)

ICG-FA, indocyanine green fluorescence angiography.

### Postoperative outcomes

The length of stay in hospital (Table 4) was similar in both groups, with a median of nine days in the ICG-FA group (IQR 10) and nine days in the control group (IQR 8) (*p*=0.092). The length of stay in the ICU (Table 4) favored the control groups with shorter ICU admission periods. The median ICU admission was four days (IQR 2.25) in the control groups and seven days (IQR 8.25) in the ICG-FA group (*p*=0.1642).

**Table 4:** Postoperative outcomes—length of stay.

	ICG-FA	Control	*p*
Length of hospital stay (median/IQR)	9 (10)	7 (8.25)	0.092
Length of ICU stay (median/IQR)	9 (8)	4 (2.25)	0.1642

ICG-FA, indocyanine green fluorescence angiography.

The ICG-FA group had significantly lower rates of postoperative complications than the control group (Table 5): 8 (40%) and 53 (66.6%) patients in the ICG-FA and control groups, respectively (*p*=0.0412). Interventions or reoperations under general anesthesia were required in two (10%) and 14 (17.5%) patients in the ICG-FA and control groups, respectively (OR 0.524, *p*=0.516). Mortality was recorded in one case (5%) in the ICG-FA group and eight out of 80 patients (10%) in the control group (*p*=0.6827). The anastomotic leak rate was lower in the ICG-FA group but not significantly lower (*p*=0.5799). It is important to note that there were no anastomotic leaks in the ICG-FA group, compared to 5 cases (6.3%) in the control group. The ICG-FA group had lower, yet not significantly lower, rates of superficial surgical site infection (OR 0.548, *p*=1), wound dehiscence of the skin (OR 0.00, Fisher exact test 0.5809), wound dehiscence of the sheath (OR 0.00, *p*=0.5809), and prolonged ileus (OR 0.2982, *p*=0.3333). The initial surgical decision was changed for eight cases (40%) following ICG-FA assessment and resection points were altered by 0.5–3.0 cm.

**Table 5:** Summary of complications.

	ICG-FA, *n* (%)	Control, *n* (%)	Odds ratio	*p*
Surgical site infection - superficial	1 (5)	7 (8.8)	0.5489	1
Surgical site infection – intra-abdominal collections	2 (10)	6 (7.5)	1.3704	0.6586
Surgical site infection - retroperitoneal fasciitis	1 (5)	1 (1.3)	4.1579	0.3616
Anastomotic leak		5 (6.3)	0.0000	0.5799
Wound dehiscence—skin		4 (5)	0.0000	0.5809
Wound dehiscence—sheath		4 (5)	0.0000	0.5809
Bile leak	1 (5)	1 (1.3)	4.1579	0.3616
Ileus—prolonged	1 (5)	12 (15)	0.2982	0.4561
Ventilator-associated pneumonia		1 (1.3)	0.0000	1
Enteric fistula—atmospheric		2 (2.5)	0.0000	1
Urine leak		2 (2.5)	0.0000	1
Death	1 (5)	8 (10)	0.4737	0.6827
Pancreatic fistula	1 (5)			0.2
Total/overall complications	8 (40)	53 (66.3)	0.3396	0.0412

ICG-FA, indocyanine green fluorescence angiography.

Minimally invasive techniques such as percutaneous catheter drainage of collections were also required more often in the control group (OR=0.0 *p*=1.0), 0 and 3 cases (3.75%) in the ICG-FA and control groups, respectively.

## Discussion

There is a vast body of research investigating the management of gastrointestinal injuries^7,11,26^. The surgical management of these injuries has evolved, from diversion being the safe alternative to primary repair or resection with anastomosis in the appropriate patient^7^.

Anastomotic leakage and its associated complications can be devastating for both the patient and the clinician. It poses an extra burden on healthcare systems and is associated with the need for further intervention^15^. We identified many risk factors in our sample that are associated with anastomotic failure. We had a significantly higher proportion of gunshot injuries, higher injury severity scores, tobacco use, hemodynamic instability, and extended waiting times for surgical intervention^4,18^. Surgeons should be aware of these factors and identify modalities to decrease the incidence of these complications.

Despite the safer reputation, diversion is also not without morbidity. Oosthuizen *et al.* ^7^ retrospectively compared the postoperative outcomes of anastomosis and stoma in penetrating abdominal injuries. They found that ICU stay was shorter in patients without a stoma, and there were more patients with postoperative complications in the diverted group. Complications following colostomy were high output stoma, necrosis, and or retraction of the stoma, ileus, fistula, and suture line failure. These stomas are also associated with further complications at the closure of the stoma. Velmahos and colleagues reported a complication rate of 26.3% in their cohort of patients, which was directly related to the colostomy closure. These complications included wound sepsis, bowel obstructions, anastomotic leak, postoperative diarrhea, and colovesical fistula^27^. Recent research has dispelled the concept of diversion based on the anatomical location of the injury and has suggested that each injury is managed on its own merits^8^. The Eastern Association for the Surgery of Trauma, as part of a meta-analysis of penetrating colonic injuries, recommended that colonic repair or resection with anastomosis be performed rather than routine diversion^28^.

The surgeon needs to balance the two modalities of anastomosis and diversion to choose the best intervention for the patient. However, this can be a challenging task. Laparotomy with ICG-FA is one such potential adjunctive modality. However, the utility of ICG-FA in the clinical setting of hemodynamic shock and poor visceral perfusion is largely unknown. A narrative review of the literature revealed the usage of ICG in trauma for a wide range of indications, including musculoskeletal trauma. The authors cited a paucity of evidence in the field of traumatic gastrointestinal injuries^29^.

The significantly lower rates of overall complications in the ICG-FA group bodes well for using ICG-FA as a surgical adjunct to improve postoperative outcomes. It is remarkable to note that there were zero anastomotic leaks in the ICG-FA group, although this was not a significantly lower result when compared to the control group. Surgeons who used ICG-FA were allowed to modify their resection margins based on ICG-FA results, and 40% of patients had their surgical plan altered based on the ICG-FA assessment. Larger prospective randomized trials may be able to clarify whether this phenomenon of zero anastomotic leaks is related to the modification of the resection points prompted by ICG-FA or to the sample size. This rate of modification of the initial surgical plan is comparable to that in a study conducted in the United States Military, in which it noted that three of nine operations (33.3%) for gastrointestinal injury had undergone modifications to the initial surgical plan^30^. In a study of ICG-FA in acute mesenteric ischemia, surgeons modified their surgical approach in 11.5% of cases.

Osterkamp and colleagues evaluated the usage and usability of ICG-FA in the emergency setting of penetrating gastrointestinal trauma. Surgeons involved in the study found the use of ICG-FA to be feasible, with increasing self-reported usability scores correlating with an increasing frequency of use^24^. Indocyanine green fluorescence angiography has also been previously described in a case series of three patients in the setting of abdominal trauma; they found the system feasible, as well as a change in the surgical resection lines^31^.

Anastomotic leakage often necessitates emergency re-laparotomy with revision or exteriorization of the injury. Revision surgery carries a high risk of iatrogenic intestinal injuries and may be impossible due to the so-called ‘frozen abdomen’^14,15^. There is also the additional risk of another anesthesia and the surgical risks associated with revision surgery. The need for interventions to address postoperative complications places an extra burden on an already-overburdened healthcare system. Revision surgery and percutaneous catheter drainage on collections were required less frequently in the ICG-FA group. The control group had two complications of entero-atmospheric fistulae. Depending on the site of the fistula, patients with these complications often have longer lengths of hospital stay and need multidisciplinary care.

The future use of ICG-FA in the assessment of gastrointestinal perfusion is ever-expanding. Quantitative indocyanine green fluorescence angiography involves the objective evaluation of intestinal perfusion by quantifying the fluorescence signals^32^. It guides surgeons of varied experience to predict resection points and thus perform safer intestinal resections^33^.

### Limitations

The single-center nature of this study, small sample size with lack of randomization, and multiple surgeons are limitations of this study. The higher cost of using ICG-FA posed an obstacle to recruiting a larger sample size. An additional limitation is the potential for selection bias, as more complex or unstable cases would deter surgeons from becoming accustomed to using this new modality. The described limitations make it difficult to draw specific conclusions and should be addressed in future larger prospective randomized trials.

## Conclusion

The use of ICG-FA can reduce overall complications following laparotomy for penetrating abdominal trauma with gastrointestinal injuries. Postoperative complications are debilitating and strain the already-overburdened healthcare system. Identifying adjuncts to reduce the risk of postoperative complications in these high-risk groups would contribute to better outcomes. Intraoperative ICG-FA may assist the surgeon in altering the surgical plan, resulting in fewer postoperative complications, as shown in our study cohort. However, our smaller sample size makes it difficult to draw specific conclusions. Despite the small sample size, the lower rate of postoperative complications in the group that underwent laparotomy with ICG-FA enhancement is promising. It suggests that larger prospective randomized studies are needed to clarify the correlation between ICG-FA and specifically anastomotic-related complications.

## Ethical approval

Stellenbosch University (Cape Town, South Africa) Health Research Ethics Committee approval: S24/03/069_Sub Study N19/08/106.

## Consent

Written informed consent was obtained from patients undergoing laparotomy with indocyanine green fluorescence angiography.

## Source of funding

No sources of funding.

## Author contribution

M.Q.P.: study concept and design, data collection, data analysis, writing of the manuscript. T.R.F.: study concept and design, writing of the manuscript, writing of the manuscript. J.J.P.B.: writing of the manuscript. J.T.F.O.: review of the manuscript. E.S.: study concept, writing of the manuscript.

## Conflicts of interest disclosure

The authors do not have any conflicts of interest.

## Research registration unique identifying number (UIN)

clinicaltrials.gov (ID:NCT04534816, https://clinicaltrials.gov/ct2/show/NCT04534816).

## Guarantor

Mohamed Quraish Patel.

## Data availability statement

The data provided here are accurate to the best of our knowledge. There have been no breaches of confidentiality.

## Provenance and peer review

Paper was not invited.

## Statements and declarations

The authors declare that they have no affiliations with or involvement in any organization or entity with any financial interests in the subject matter or materials discussed in this manuscript.
